# Data set on G4 DNA interactions with human proteins

**DOI:** 10.1016/j.dib.2018.02.081

**Published:** 2018-03-09

**Authors:** M. Vlasenok, O. Levchenko, D. Basmanov, D. Klinov, A. Varizhuk, G. Pozmogova

**Affiliations:** Research and Clinical Center for Physical Chemical Medicine, 119435 Moscow, Russia

## Abstract

Guanine-rich DNA/RNA fragments can fold into G-quadruplexes (G4s) – non-canonical four-strand secondary structures. The article contains data on quadruplex interaction with human proteins. Binding of three topologically different G4 structures to more than 9000 human proteins was analyzed. Physicochemical methods were used to verify the results.The dataset was generated to identify the protein targets for DNA quadruplex structures for the purpose of better understanding the role of the structures in gene expression regulation. Presented data include functional interpretation of obtained gene lists, visualized with Cytoscape.

**Specifications table**

**Table:** 

Subject area	Molecular Biology; Physical chemistry
More specific subject area	DNA secondary structures
Type of data	Tables, high resolution images, figures, network diagrams
How data was acquired	Small Molecule-Protein Interaction Profiling on ProtoArray® Human Protein Microarrays (Invitrogen); CD spectroscopy (JASCO V-550 spectrophotometer); fluorescence polarization measurements (Cary Eclipse fluorescence spectrophotometer, Agilent Technologies); fluorescence decay measurements (Easy Life V fluorescence lifetime fluorometer, Optical Building Blocks Corporation), electrophoretic mobility shift assay (results were visualized using Gel Doc XR+ system BIO RAD)
Data format	Raw, analyzed
Experimental factors	ON solutions in the specified buffers were denatured at 95 °C for 5 min and snap cooled on ice prior to the experiments
Experimental features	Small Molecule-Protein Interaction Profiling was performed using ProtoArray® Human
	Protein Microarrays, CD spectroscopy was recorded on a spectrophotometer equipped with temperature-controlled cuvette holder, fluorescence rotational relaxation times were calculated using fluorescence polarization and fluorescence lifetime values, the gel in electrophoretic mobility shift assay was stained with SYBR Gold.
Data source location	Research and Clinical Center for Physical Chemical Medicine and Engelhardt Institute of Molecular Biology, Moscow, Russian Federation
Data accessibility	The data is available within this article

**Value of the data**

• These data describe the use of human protein microarrays to identify binding partners of G4 DNA structures.
• Functional enrichment analysis of G4-binding proteins can be used to elucidate biological roles of genomic G4s and possible side effects of G4-based therapeutics.
• Data allow one to compare topologically different G4s in terms of their interactions with human proteins.
• Data can be added to previously published data on G4-binding proteins for a more integrated view of the G4 interactome.

## 1 Data

**Fig. 1:**
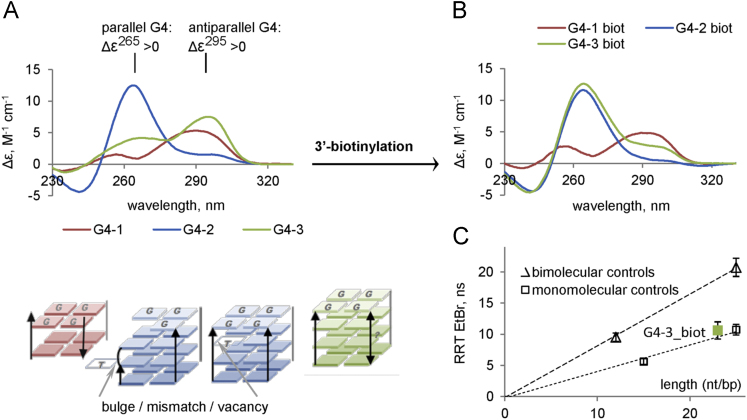
**Characterization of biotinylated G4s. (A)** СD spectra (per mole of nucleotide) and schematic representations of previously characterized initial (non-biotinylated) G4 structures. **(B)** CD spectra of the 3’-biotinylated G4s. **(C)** Rotational relaxation time (RRT) of EtBr in complex with G4-3. Conditions: 20 mM Tris–HCl buffer (pH 7.5), 100 mM KCl. Points on the graph correspond to the average values of three measurements.

**Fig. 2:**
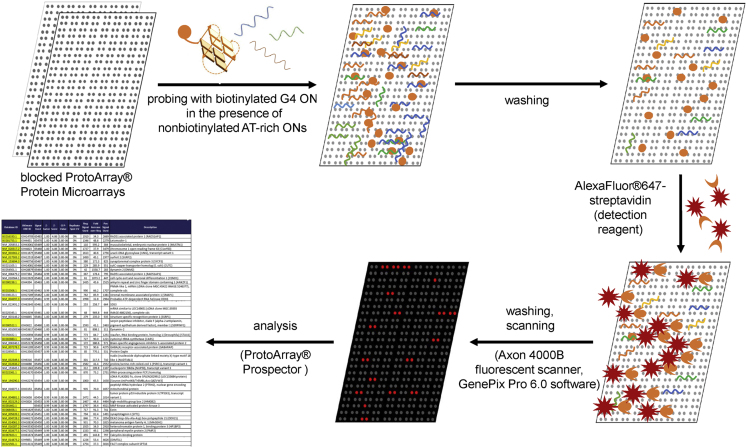
Workflow of profiling G4-protein interactions using human protein microarrays.

**Fig. 3:**
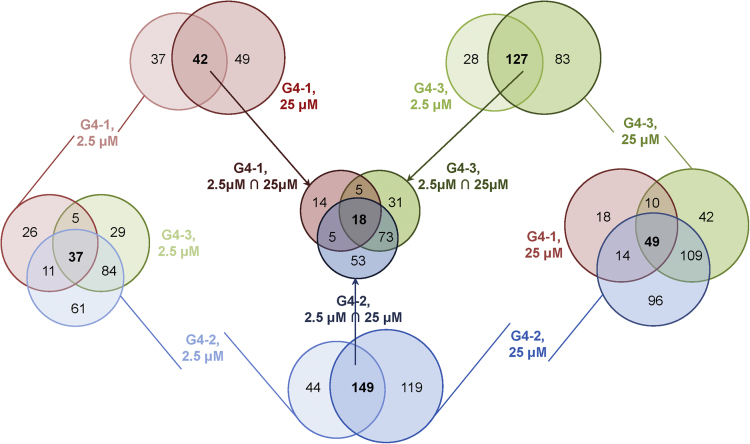
Three Venn diagrams for protein interactors of G4 ONs profiled at different concentrations.

**Fig. 4:**
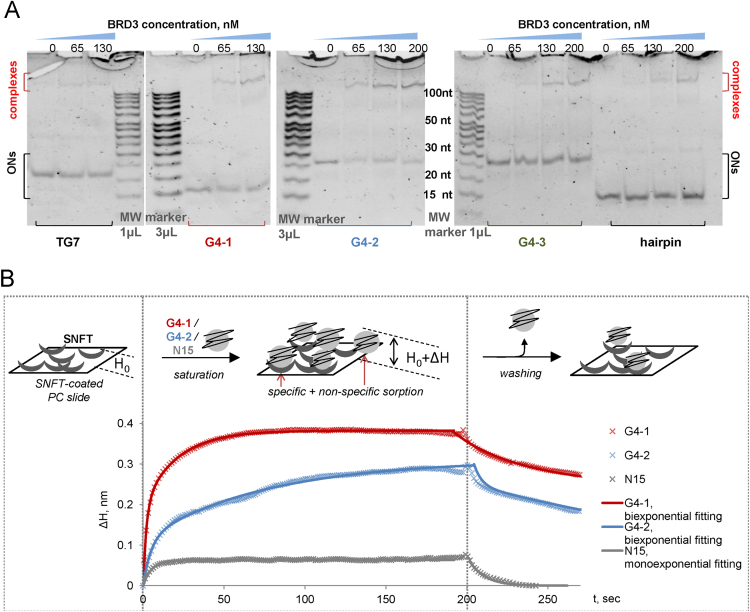
**Verification of the profiling data for selected proteins. (A)** Data on BRD3 interactions with G4 and non-G4 (hairpin and (TG)_7_) ONs: electrophoretic mobility shift assay. **(B)** Data on SNFT interactions with G4 and non-G4 (N15) ONs: photonic crystal surface wave assay. The sensorgrams illustrate changes in the effective adlayer thickness (delta H) upon ON sorption on/desorption from the SNFT-coated photonic crystal (PC) slide surface.

**Figure S1:**
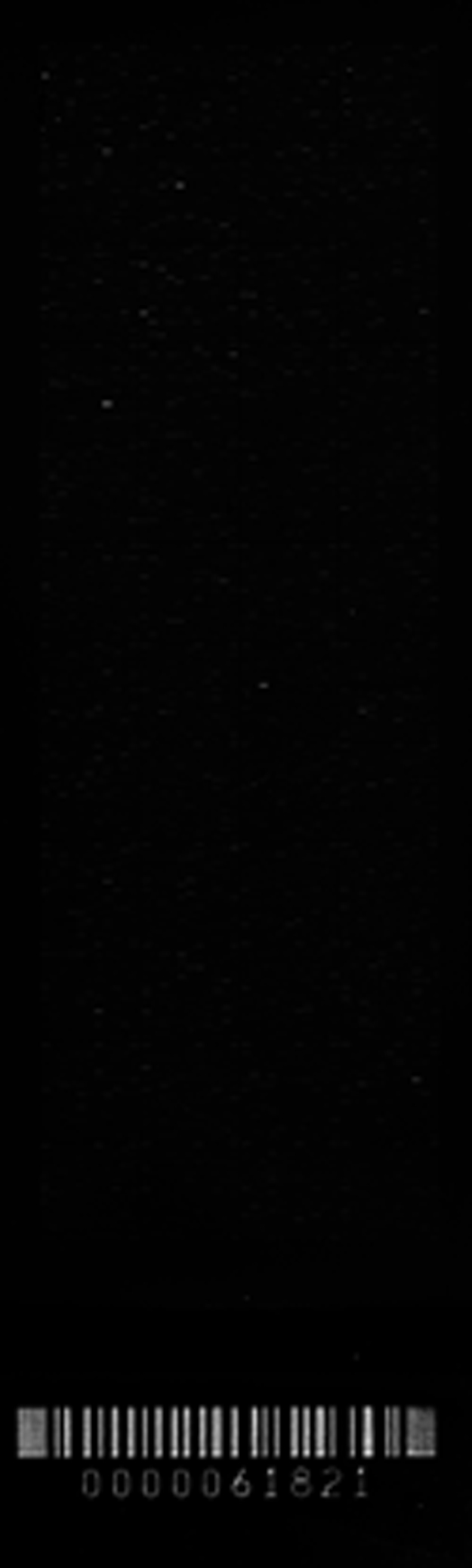
High Resolution Array Image. Positive control.

**Figure S2:**
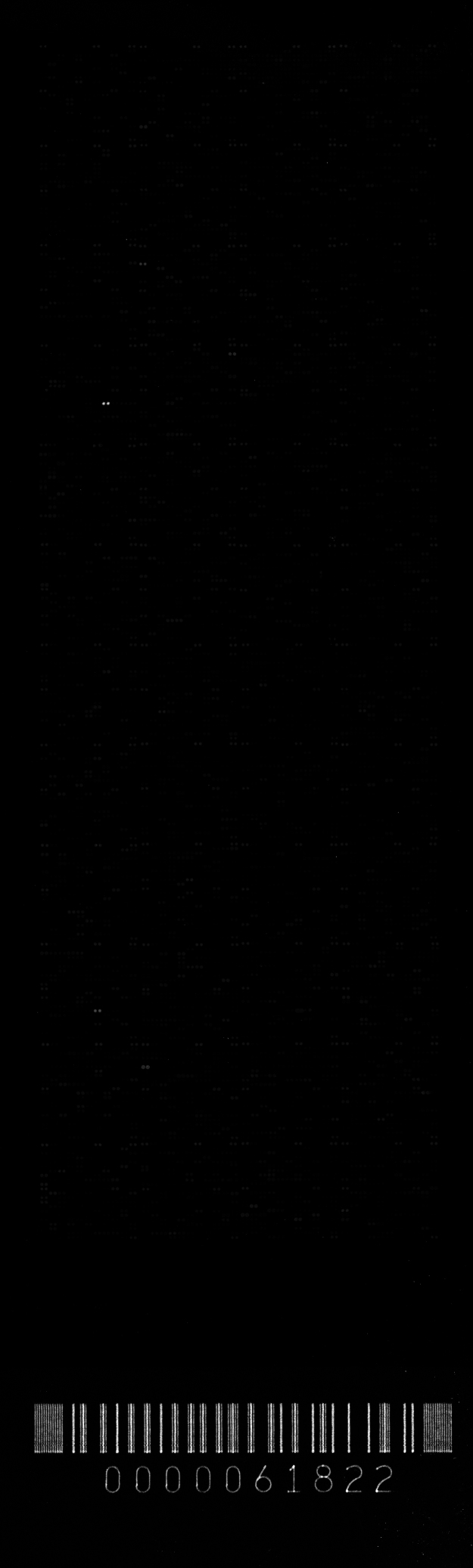
High Resolution Array Image. Negative control.

**Figure S3:**
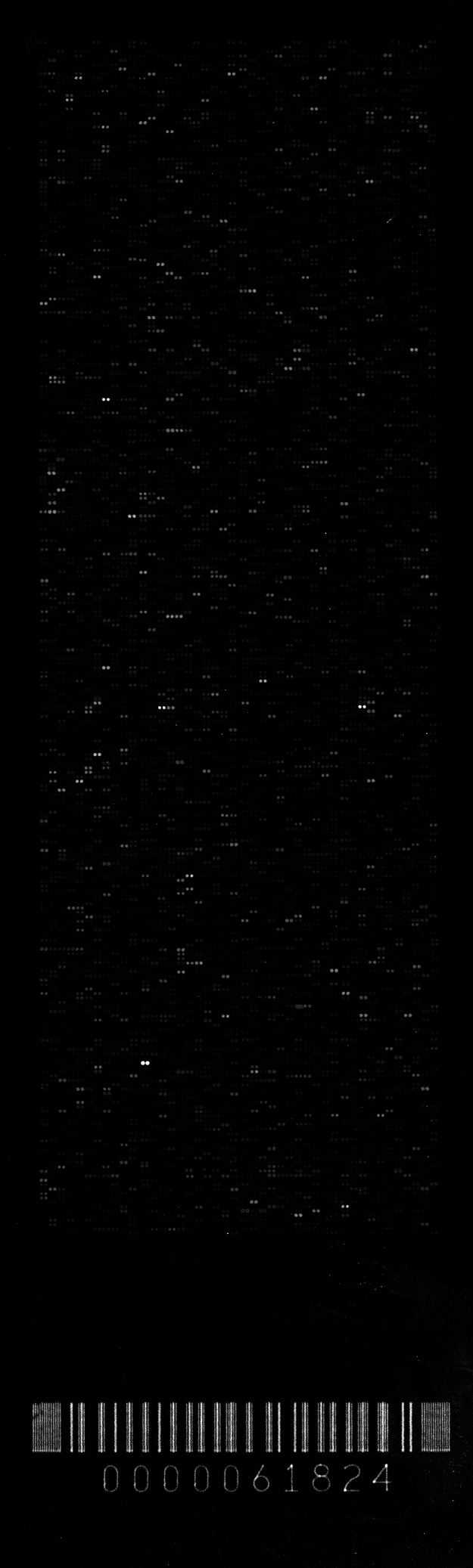
High Resolution Array Image. Experimental assay with G4-1, profiled at 2.5 μM.

**Figure S4:**
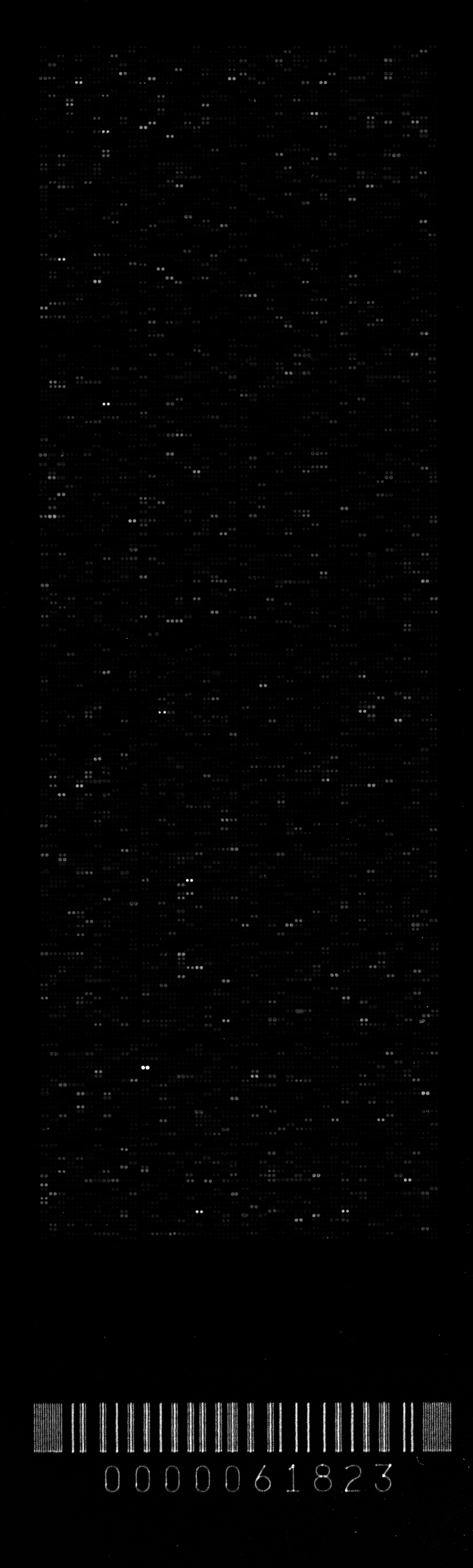
High Resolution Array Image. Experimental assay with G4-1, profiled at 25 μM.

**Figure S5:**
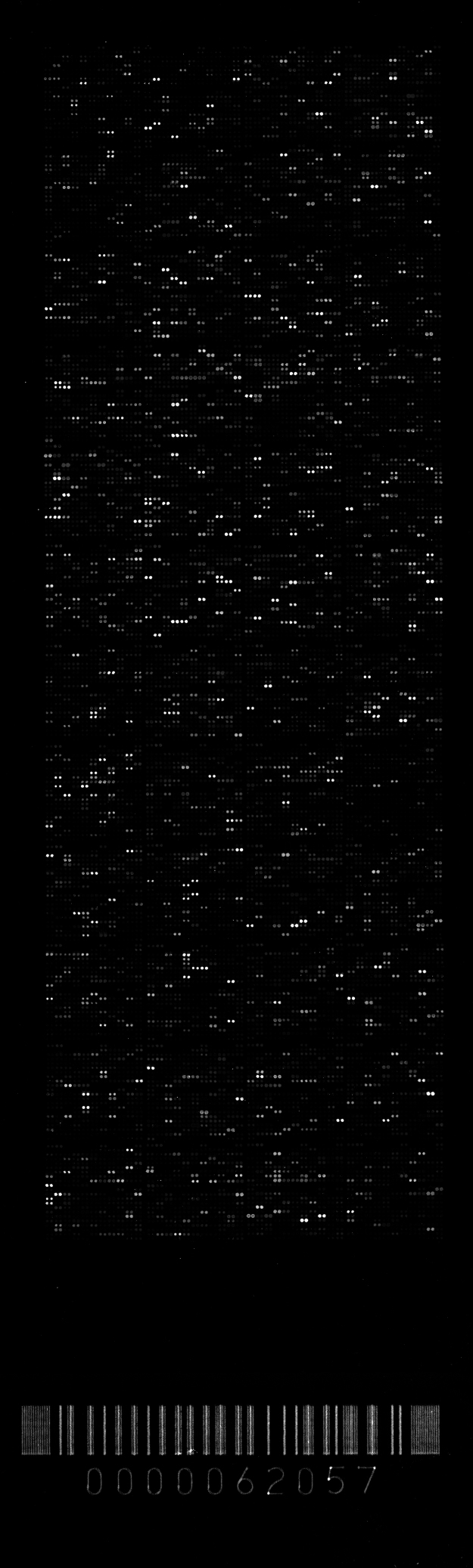
High Resolution Array Image. Experimental assay with G4-2, profiled at 2.5 μM.

**Figure S6:**
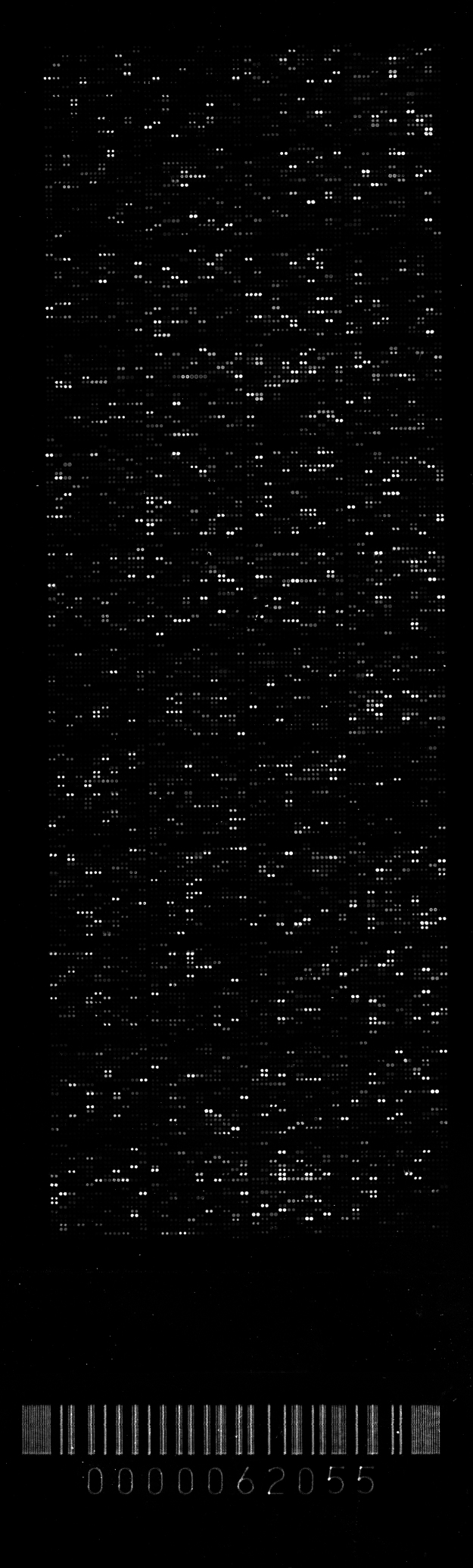
High Resolution Array Image. Experimental assay with G4-2, profiled at 25 μM.

**Figure S7:**
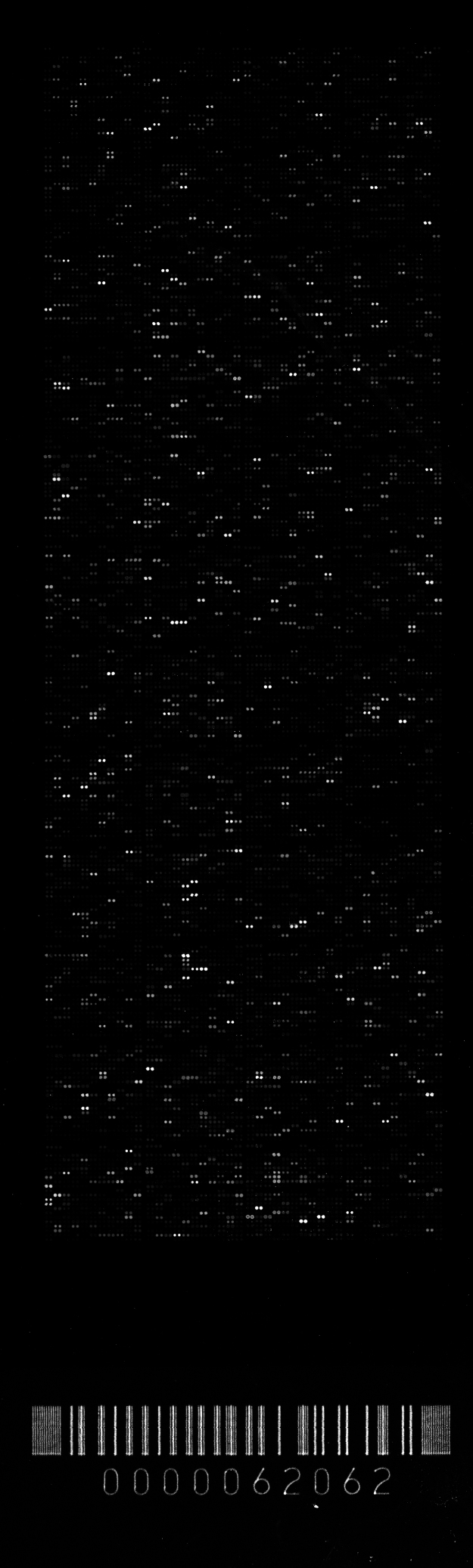
High Resolution Array Image. Experimental assay with G4-3, profiled at 2.5 μM.

**Figure S8:**
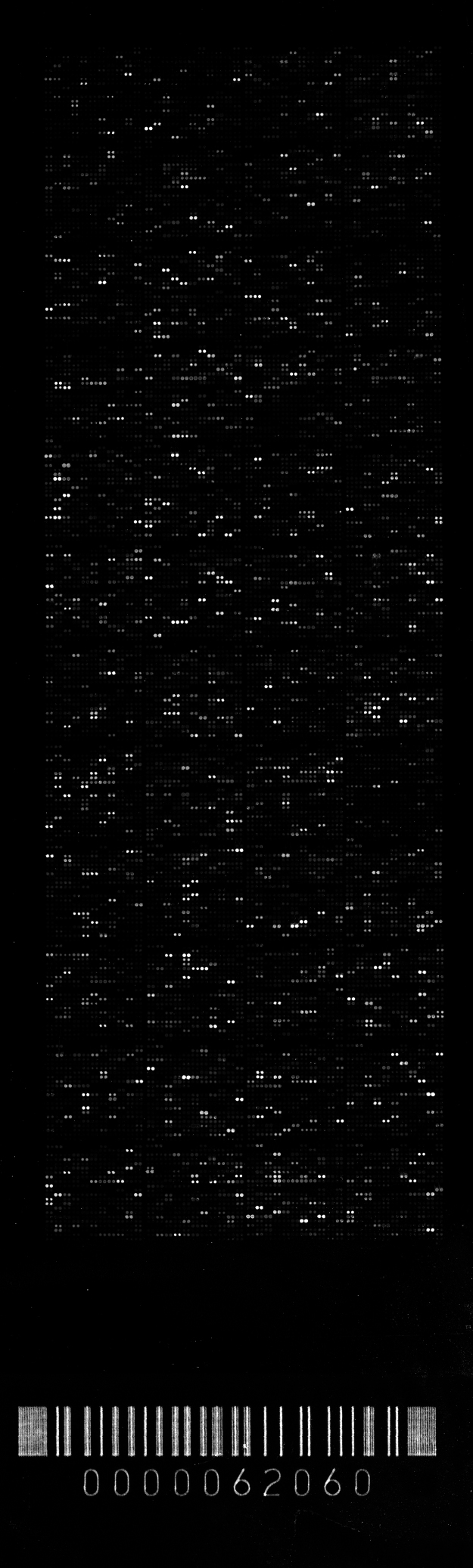
High Resolution Array Image. Experimental assay with G4-3, profiled at 25 μM.

The dataset of this article provides information on interactions between G4-forming oligodeoxyribonucleotides (G4-1, G4-2 and G4-3) and human proteins (over 9000) immobilized on microarrays. The set of oligonucleotides (ONs) was designed to allow comparison of topologically different intramolecular G4 structures. According to published circular dichroism (CD) data, G4-1 ON (d(GGGAGGCTGAGGCAGG), designated previously as PQS2 [1]) adopts antiparallel topology in the presence of potassium ions [2], G4-2 ON (d(GGTGACAGGGGTATGGGGAGGGG), designated previously as Ct1 [3]) forms a parallel quadruplex structure, and G4-3 ON (d(GGGGACAGGGGTATGGGGAGGGG), designated previously as CtG [3]) adopts mixed topology (the “antipatrallel” CD signature dominates [4]). Terminal modification (biotinylation) required for the profiling experiments could alter ON secondary structures, therefore, CD spectra of the modified ONs were recorded to verify the maintenance of G4 folding. Substantial difference from the published spectra was observed only for G4-3, so we additionally performed a rotational relaxation time assay (RRT) to verify its intramolecular folding. Characterization of the biotinylated G4s by optical methods is summarized in Fig. 1. Fig. 2 is an overview of the protein array profiling procedure. Supplementary Figures S1–8 are high resolution scans of the control protein arrays and arrays after treatment with solutions of biotinylated G4s and visualization of the interactions using streptavidin-conjugated Alexa Fluor®647 (white spots in the array scans refer to G4-protein complexes). Fig. 3 shows intersection of the sets of significant protein hits identified in the profiling experiments with each G4. Table 1, Table 2, Table 3, Table 4, Table 5 contain lists of protein hits from the central diagram in Fig. 3 and corresponding significance parameter values (Z-score). Functional interpretations of the respective gene lists visualized with Cytoscape are provided as Supplementary networks 1–7. Supplementary files SF1 and SF2 contain data on all investigated proteins, their parameters and interactions with each G4. Fig. 4 shows verification of the profiling data for two selected proteins by electrophoretic method and a method based on photonic crystal surface waves (PCSW).

**Table 1:** Top non-specific protein hits. Overlap of the three G4 interactor sets significant at both 2.5 μM and 25 μM G4 concentrations, ranked by average Z-score (G4-1 **∩** G4-2 ∩ G4-3). TV = transcript variant.

**Protein**	**Database ID**	**Z-score, 2.5 μM / 25 μM**		
		**G4-1**	**G4-2**	**G4-3**
hypothetical protein HSPC111 (HSPC111)	BC040106.1	7.99 / 6.30	6.78 / 4.85	9.11 / 6.46
additional sex combs like 1 (Drosophila) (ASXL1)	BC064984.1	6.13 / 7.31	6.79 / 4.86	9.22 / 6.47
***bromodomain containing 3 (BRD3)***	BC032124.1	8.82 / 9.28	6.79 / 4.85	4.51 / 6.47
chromosome 1 open reading frame 63 (C1orf63)	NM_020317.2	5.41 / 10.51	6.17 / 4.86	5.93 / 6.47
cyclin B3 (CCNB3), transcript variant 2	NM_033671.1	7.96 / 5.45	6.74 / 4.81	6.97 / 6.42
peptidyl arginine deiminase, type IV (PADI4)	NM_012387.1	5.72 / 6.58	6.78 / 4.85	7.58 / 5.27
Coiled-coil domain-containing protein 28 A	BC000758.1	4.95 / 4.88	6.78 / 4.85	8.15 / 6.45
ankyrin repeat and zinc finger domain containing 1 (ANKZF1)	BC000238.1	6.13 / 4.46	6.68 / 4.86	6.34 / 6.15
GABA(A) receptor-associated protein (GABARAP)	NM_007278.1	4.42 / 5.13	6.62 / 4.86	7.02 / 6.47
Cyclin-dependent kinase-like 3	NM_016508.2	6.26 / 4.83	6.29 / 4.86	5.63 / 6.20
actin related protein 2/3 complex, subunit 1B, 41 kDa (ARPC1B)	NM_005720.1	5.47 / 5.10	6.19 / 4.86	6.54 / 5.66
peripheral myelin protein 2 (PMP2)	NM_002677.1	4.12 / 3.49	6.73 / 4.86	7.28 / 6.31
Peptidyl-tRNA hydrolase 2, mitochondrial	NM_001015509.1	6.85 / 6.94	3.46 / 4.85	3.95 / 4.81
survival motor neuron domain containing 1 (SMNDC1)	BC011234.1	4.49 / 3.60	6.40 / 4.86	4.79 / 6.46
small trans-membrane and glycosylated protein (LOC57228), transcript variant 2	NM_020467.2	3.31 / 4.28	5.81 / 4.86	4.59 / 5.78
La ribonucleoprotein domain family, member 1 (LARP1)	BC033856.1	4.49 / 4.62	5.37 / 4.85	4.71 / 4.09
cDNA FLJ42001 fis, clone SPLEN2029912 (LOC153684 protein)	NM_194290.1	3.85 / 3.39	6.45 / 4.86	4.46 / 4.40
splicing factor, arginine/serine-rich 6 (SFRS6)	NM_006275.2	4.01 / 4.49	3.80 / 3.50	5.18 / 3.18

**Table 2:** Top semi-specific protein hits. Pairwise overlaps of the G4 interactor sets significant at both G4 concentrations, ranked by average Z-score. TV = transcript variant.

**Protein**	**Database ID**	**Z-score, 2.5 μM / 25 μM**	
**G4-1 ∩G4-2**			
		**G4-1**	**G4-2**
MAP kinase-activated protein kinase 3	BC001662.1	6.79 / 4.86	6.60 / 5.98
RNA polymerase II-associated protein 3	BC056415.1	6.32 / 4.62	4.70 / 6.19
***Jun dimerization protein p21SNFT (SNFT)***	NM_018664.1	6.00 / 3.68	4.53 / 3.81
Band 4.1-like protein 4 A	NM_022140.2	4.23 / 4.85	3.59 / 3.55
rRNA-processing protein FCF1 homolog	BC022361.1	4.28 / 4.86	3.18 / 3.88

**G4-1 ∩G4-3**			
		**G4-1**	**G4-3**
Finkel-Biskis-Reilly murine sarcoma virus (FBR-MuSV) ubiquitously expressed (FAU)	NM_001997.2	6.01 / 5.79	8.88 / 6.47
nucleolar protein 7, 27 kDa (NOL7)	NM_016167.3	4.76 / 5.88	8.76 / 6.47
PHD finger protein 20-like 1 (PHF20L1), TV 3	NM_198513.1	5.84 / 5.57	7.63 / 6.47
Ras-like without CAAX 1 (RIT1)	NM_006912.3	6.00 / 3.83	6.28 / 5.58
casein kinase 2, alpha 1 polypeptide (CSNK2A1), TV 1	NM_177559.2	5.06 / 4.01	5.58 /4.25

**G4-2 ∩G4-3 (top 20)**			
		**G4-2**	**G4-3**
fibroblast growth factor 12 (FGF12)	BC022524.1	6.79 / 4.85	8.31 / 6.47
DIM1 dimethyladenosine transferase 1-like (S. cerevisiae) (DIMT1L)	NM_014473.2	6.79 / 4.86	8.31 / 4.84
Probable ATP-dependent RNA helicase DDX6	NM_004397.3	6.79 / 4.86	7.51 / 5.57
fibroblast growth factor 12 (FGF12), TV 1	NM_021032.2	5.82 / 4.85	8.02 / 5.89
Non-histone chromosomal protein HMG-14	BC070154.1	6.03 / 4.77	7.64 / 6.04
FACT complex subunit SPT16	BC021561.1	6.76 / 4.86	6.59 / 6.02
heterochromatin protein 1, binding protein 3 (HP1BP3)	NM_016287.2	6.79 / 4.86	6.82 / 5.03
KRR1, small subunit (SSU) processome component, homolog (yeast) (KRR1)	BC016778.1	6.50 / 4.85	6.33 / 5.50
Nuclear protein Hcc-1	BC093051.1	4.43 / 4.85	7.27 / 6.41
RAD51 associated protein 1 (RAD51AP1)	BC016330.1	6.79 / 4.86	4.69 / 6.18
Serine/threonine-protein kinase 12	BC000442.1	6.15 / 4.86	5.47 / 5.82
serpin peptidase inhibitor, clade F (alpha-2 antiplasmin, pigment epithelium derived factor), member 1 (SERPINF1)	BC000522.1	5.71 / 4.86	5.21 / 6.47
high-mobility group box 2 (HMGB2)	NM_002129.2	6.19 / 4.86	7.26 / 3.68
high mobility group nucleosomal binding domain 3 (HMGN3), TV 2	NM_138730.1	4.29 / 4.86	6.66 / 6.10
small proline-rich protein 4 (SPRR4)	NM_173080.1	6.44 / 4.86	5.25 / 5.18
Regulator of G-protein signaling 3	BC019039.2	5.68 / 4.85	5.25 / 5.59
RAB35, member RAS oncogene family (RAB35)	NM_006861.2	5.92 / 4.54	5.81 / 5.09
G protein-coupled receptor kinase 6	NM_002082.1	5.15 / 4.86	6.21 / 5.13
Ras-like without CAAX 2 (RIT2)	BC018060.1	4.81 / 4.85	5.52 / 6.07
signal transducing adaptor family member 1 (STAP1)	NM_012108.1	5.96 / 3.96	6.03 / 5.01

**Table 3:** G4-1-specific/semi-specific protein hits. Significant interactors of G4-1 at both concentrations classified as insignificant for G4-2 and G4-3 at one or both concentrations (the non-overlapping subset of the G4-1 interactor set, ranked by average Z-score). TV = transcript variant.

**Protein**	**Database ID**	**Z-score**	
		**2.5 μM G4-1**	**2.5 μM G4-1**
Regulator of G-protein signaling 3 (RGS3), TV 4	NM_134427.1	7.50	8.19
OTU domain containing 6B (OTUD6B)	BC029760.1	8.13	7.08
Mitogen-activated protein kinase-activated protein kinase 5 (MAPKAPK5), TV 1	NM_003668.2	6.58	7.71
Casein kinase 2, alpha 1 polypeptide (CSNK2A1), TV 2	NM_001895.1	6.10	6.85
Chromatin modifying protein 6 (CHMP6)	NM_024591.1	7.01	4.39
Chromosome 11 open reading frame 63 (C11orf63), TV 2	NM_199124.1	4.93	6.17
Mitochondrial ribosomal protein L19 (MRPL19), nuclear gene encoding mitochondrial protein	NM_014763.2	4.58	4.49
Chondrosarcoma associated gene 1 (CSAG1)	BC059947.1	4.93	3.83
La ribonucleoprotein domain family, member 6 (LARP6), TV 1	NM_018357.2	3.99	4.70
Dihydrouridine synthase 1-like (S. cerevisiae) (DUS1L)	NM_022156.3	3.49	3.75
Coiled-coil domain containing 23 (CCDC23)	BC029427.1	3.60	3.52
PRKR interacting protein 1 (IL11 inducible) (PRKRIP1)	BC014298.1	3.60	3.47
UBX domain containing 3 (UBXD3)	NM_152376.2	3.52	3.24
LSM4 homolog, U6 small nuclear RNA associated (S. cerevisiae) (LSM4)	NM_012321.1	3.24	3.26

**Table 4:** G4-2-specific/semi-specific protein hits (top 20). Significant interactors of G4-2 at both concentrations classified as insignificant for G4-1 and G4-3 at one or both concentrations (the non-overlapping subset of the G4-2 interactor set, ranked by average Z-score). TV = transcript variant.

**Protein**	**Database ID**	**Z-score**	
		**2.5 μM G4-2**	**2.5 μM G4-2**
Nuclear protein Hcc-1	NM_033082.1	6.78	4.85
PNMA-like 1, mRNA (cDNA clone MGC:45422 IMAGE:5246377), complete cds	BC032508.1	6.09	4.86
chromosome 11 open reading frame 52 (C11orf52)	NM_080659.1	6.00	4.71
Ubiquitin specific peptidase 39 (USP39)	NM_006590.2	5.86	4.83
Hypothetical protein MGC31957 (MGC31957)	BC005043.1	5.59	4.86
Small inducible cytokine subfamily E, member 1 (endothelial monocyte-activating) (SCYE1)	BC014051.1	5.44	4.85
Mitochondrial ribosomal protein S6 (MRPS6), nuclear gene encoding mitochondrial protein	NM_032476.1	5.37	4.60
Synaptotagmin I (SYT1)	NM_005639.1	4.95	4.86
Cysteinyl-tRNA synthetase (CARS)	BC002880.1	4.70	4.86
Regulator of G-protein signaling 8	BC069677.1	4.59	4.85
Coiled-coil domain containing 43 (CCDC43)	BC047776.2	4.18	4.85
Rho GTPase-activating protein 12	BC094719.1	4.46	4.54
Ring finger protein 4 (RNF4)	NM_002938.2	4.10	4.86
Within bgcn homolog (Drosophila) (WIBG)	NM_032345.1	4.08	4.85
Proline/serine-rich coiled-coil 1 (PSRC1), TV 1	NM_032636.2	4.05	4.86
Nucleophosmin (nucleolar phosphoprotein B23, numatrin) (NPM1)	BC021983.1	3.73	4.85
Protein FAM76B	NM_144664.3	4.14	4.39
Spermatogenesis associated, serine-rich 2 (SPATS2)	BC048299.1	3.78	4.63
Synaptonemal complex protein 3 (SYCP3)	NM_153694.3	3.54	4.86
Ezrin	BC068458.1	3.51	4.86

**Table 5:** G4-3-specific/semi-specific protein hits (top 20). Significant interactors of G4-2 at both concentrations classified as insignificant for G4-1 and G4-3 at one or both concentrations (the non-overlapping subset of the G4-2 interactor set, ranked by average Z-score). TV = transcript variant.

**Protein**	**Database ID**	**Z-score**	
		**2.5 μM G4-3**	**2.5 μM G4-3**
Methionyl aminopeptidase 2 (METAP2)	NM_006838.1	8.14	6.08
Potassium channel tetramerisation domain containing 18 (KCTD18)	BC067755.1	3.35	6.17
GTPase activating protein (SH3 domain) binding protein 1 (G3BP1), transcript variant 2	NM_198395.1	4.67	4.78
CAP-GLY domain containing linker protein family, member 4 (CLIP4)	NM_024692.3	4.34	4.82
Polymerase (DNA directed), beta (POLB)	NM_002690.1	5.02	4.00
Chromosome 6 open reading frame 130 (C6orf130)	NM_145063.1	5.72	3.18
Transcription elongation factor A (SII)-like 2 (TCEAL2)	NM_080390.3	4.59	4.10
Laminin, gamma 1 (formerly LAMB2) (LAMC1)	BC015586.2	3.93	4.23
Three prime histone mRNA exonuclease 1 (THEX1)	NM_153332.2	4.03	4.09
Ribosomal protein L35 (RPL35)	BC010919.1	3.25	4.72
RNA (guanine-9-)-methyltransferase domain-containing protein 3	BC057774.1	4.57	3.36
Angiogenic factor with G patch and FHA domains 1	BC029382.1	4.00	3.80
Chromosome 8 open reading frame 59 (C8orf59)	BC032347.1	3.45	4.32
Membrane protein, palmitoylated 7 (MAGUK p55 subfamily member 7) (MPP7)	BC038105.2	3.33	4.39
Cyclin-dependent kinase-like 1	NM_004196.2	4.29	3.32
Double-stranded RNA-binding protein Staufen homolog 1	NM_004602.2	3.65	3.91
Transcription factor AP-2 beta (activating enhancer binding protein 2 beta) (TFAP2B)	NM_003221.1	3.73	3.40
Methyltransferase like 1 (METTL1), transcript variant 1	NM_005371.2	3.98	3.14
Cell division cycle 7-related protein kinase	NM_003503.2	3.46	3.56
Histone cluster 2, H2ac (HIST2H2AC)	NM_003517.2	3.91	3.01

## 2 Experimental design, materials and methods

### 2.1 G4 design, synthesis and characterization by optical methods

Sequences of ONs G4-1 and G4-2 were chosen as well-characterized quadruplex motifs from the human genome. G4-1 is an Alu repeat fragment, the only potential quadruplex site in a conservative region of Alu elements (PQS2 in [1]). G4-2 is a fragment of CTIF gene intron (NCBI Reference Sequence: NC_000018.9, chr18: +46379322 to +46379344), it reportedly forms an “imperfect” quadruplex structure with a bulge between G-tetrads or a mismatch/vacancy in a tetrad (Ct1 in [3]). G4-3 is a G4-2 mutant, it forms a canonical (“perfect”) G4 structure (CtG in [3]). Standard solid-phase ON synthesis was performed using standard reagents and 3’-BiotinTEG CPG (Glen Research). HPLC purification and MALDITOF MS analysis were performed as previously described [4]. The purity of all oligonucleotides was determined to be ≥95% by HPLC.

Circular dichroism spectra of the 3’-biotin-ONs in 20 mM Tris–HCl buffer (pH 7.5) containing 100 mM KCl were recorded using a V-550 spectrophotometer (JASCO). ON concentration was 3 μM. The samples were heated to 90 °C for 5 minutes and cooled rapidly prior to the experiments. Molar CD per nucleotide residue was calculated as follows: Δε = θ/(32982∙C∙n∙l), where θ is ellipticity (degree), C is ON concentration (M), l is optical path length (cm) and n is the number of nucleotide residues in the ON.

Rotational relaxation times (RRT) of ethidium bromide (EtBr) in complexes with the ONs were estimated to clarify whether ON G4-3 folds into an inter- or intramolecular structure (in the first approximation RRT is proportional to the hydrodynamic volume of the complex). RRT was calculated based on EtBr fluorescence lifetime and polarization values as described in [4]. The fluorescence lifetime was evaluated using Easy Life V (fluorescence decay was registered through a RG610 long pass filter at excitation LED 525 nm). Fluorescence polarization was calculated based on vertical and horizontal components of fluorescence intensity at emission maximum (610 nm) that were measured with Cary Eclipse spectrofluorometer at 4 °C upon excitation at 540 nm by the vertically polarized light. Concentration of EtBr was 1 μM, and ON concentration was 5 μM. To correlate G4-3 RRT with inter/intramolecular folding, ‘monomolecular’ and ‘bimolecular’ calibration plots were obtained using previously described (control) ON structures. Monomolecular controls were d(GGGTGGGTGGGTGGG) (G3 in [3] and [4]) and d(GGGGGCCGTGGGGTGGGAGCTGGGG) (Bcl in [3] and [4]). Bimolecular controls were d(TCACCTCCCTCC/ GGAGGGAGGTGA) (duplex) and d(GGGGGCCGTGGGGTGAGAGCTGGGG) (BclA in [3] and [4]).

### 2.2 Small molecule - protein interaction assays

To identify G4-binding proteins, we used the commercially available profiling service “Biotinylated or fluorophore-labeled small molecule detection-identification of fluorescent or biotinylated drug target substrates”. The profiling experiments were performed on protein microarrays containing duplicate probes of more than 9000 human proteins and additional control proteins spotted on a modified glass slide by Invitrogen (ProtoArray® Human Protein Microarray Version 5.0; Invitrogen http://www.invitrogen.com). Three biotinylated ONs (G4-1, G4-2 and G4-3) were profiled at two concentrations (2.5 μM and 25 μM). The ON solutions in a working buffer (20 mM Tris (pH 7.5), 5 mM MgCl_2_, 100 mM KCl) were annealed rapidly prior to the experiments as described in the previous subsection to ensure intramolecular folding, and 0.1% Tween-20 was added to each sample after annealing to prevent aggregation. A mixture of non-biotinylated A/T-rich ONs was used to block non-specific interactions in the profiling experiments. The mixture consisted of random-sequence ONs (N_6_, N_10_ and N_15_) and AT-rich ONs (listed below) in the ratio 2:3.

A/T-rich ONs in the mixture: d(gCTTCTTgCCgAgATTTCgATTACTACCATTTTTTTTTTCATgC); d(gggATACTgATgACTgCCAgC); d(AgAAATCTCAgTTCCTACAgA); d(ggCgCTggCTTgACAgTTTC); d(CTgCCTTCAggTTgTTgCTTg); d(TATCCAgCTTTCTgTAACTTC); d(TCCCTAggTATCTggAATAg); d(gCAgAgACATCTTAACTTCAC); d(TgAgTTTCACAAAgCTATCTg); d(TAATTgAgATggAggTATgTC); d(TTACATAgCTgggAggATATC); d(TTTATCACCAAgTgCATgAAg); d(AATTTACTATATAAATTATACTAATCCA).

Two types of control assays (negative and positive) were performed on separate ProtoArray® Human Protein Microarrays in parallel with assays probed with G4s (detailed information about control assays is available from the Invitrogen web-page page [5]).

The data on G4-protein interactions were processed by ProtoArray® Prospector (the manual is available to the general public). Significance call queries were performed by Prospector to identify hits on each protein array. Significance score (Z-score) was calculated as difference between signal minus background values for the protein of interest and mean signal minus background value for all non-control proteins on the array, divided by the standard deviation of the human protein features. The protein has positive significance call if corresponding Z-score is greater than 3.0. Interactors (hits) are defined as proteins having positive significance calls that are not observed for the appropriate negative control.

### 2.3 Verification of the microarray profiling data

To verify the results of the microarray experiments, two proteins (BRD3 and SNFT) were selected from the top ‘non-specific’ and ‘semi-specific’ hits (Table 1, Table 2, respectively; Italics), and their interactions with G4s were assessed by independent methods. Recombinant BRD3 and SNFT were purchased from and Abnova(Taiwan), #H00008019-P01 and MyBioSource(USA), #MBS953061, respectively.

G4 interactions with BRD3 were assessed by electrophoretic mobility shift assay. Non-G4 ssDNA (TG)_7_ and hairpin DNA d(TCACATTCCTGAGAATGTGA) were used as controls to address the specificity factor. Preannealed ON solutions (200 nM) in 20 mM Tris–HCl buffer (pH 8) containing 100 mM KCl were incubated with recombinant protein BRD3 (concentrations: 0–200 nM) for 5 min at room temperature. The band shifts were resolved on a 15% non-denaturing polyacrylamide gel (29:1) in a standard TBE buffer (pH 8). A mixture of 10–100 nt single-stranded oligonucleotide fragments was used as molecular weight marker (Low MW Marker, Affymetrix). The gel was stained with SYBR Gold and analyzed using a GelDoc scanner (BioRad).

G4 interactions with SNFT were assessed using biosensors based on photonic crystal surface waves (PCSW) [6]. A mixture of 15-mer ONs with randomized sequences (N15) was used as a control to address the specificity factor. The protein was immobilized on the functionalized photonic crystal (PC) slide surface. PC slide preparation and functionalization were performed as described in [7]. ON solutions (1.5 µM) in standard PBS buffer (10 mM sodium phosphate, pH 7.4, 140 mM NaCl, and 3 mM KCl) were pumped through the working chamber (over the slide surface), and binding was registered as an increment of the effective adlayer thickness (ΔH). After signal saturation, the chamber was rinsed with the working buffer to remove non-specifically bound ONs.

### 2.4 Gene ontology enrichment analysis

We determined which Gene Ontology (GO) categories are statistically overrepresented in the set of proteins, which interact with G4 according to microarray experiment results. Enrichment analysis of the molecular functions of quadruplex binding proteins was performed with BiNGO plugin [8] with custom ontology and annotation files derived from the Gene Ontology database, all proteins present on the Human Protein Microarray were used as a custom reference set. For assessing overrepresentation accurately Hypergeometric statistical test (test without replacement) was used with Benjamini&Hochberg False Discovery Rate (FDR) correction. The results were visualized with Cytoscape 3.6.0.

## Appendix A Supplementary material

**File SF1. Complete protoarray data.** The data presented includes the background subtracted pixel intensity value (the “Signal Used” value), the Z-Factor, Z-Score, CI P-Value as well as the coefficient of variation (CV) associated with the replicate spots for each protein. GenBank accession identifiers and a gene description are included. Candidate interactors identified at both concentrations tested are highlighted yellow.

**File SF2.** Significant protein hits for each G4 at each concentration (the hits significant at both concentrations are highlighted).

**Network 1** Molecular functions network for significant protein hits (interactors) of G4-2 and G4-3 (25 μM concentration), P value threshold = 5%.

**Network 2** Molecular functions network for significant protein hits (interactors) of G4-2 (both 2.5 and 25 μM concentrations), P value threshold = 5%.

**Network 3** Molecular functions network for significant protein hits (interactors) of G4-3 (both 2.5 and 25 μM concentrations), P value threshold = 5%.

**Network 4** Molecular functions network for significant protein hits (interactors) of G4-1 (2.5 μM concentration) binding proteins (P value threshold = 5%).

**Network 5** Molecular functions network for significant protein hits (interactors) of G4-1 (25 μM concentration) binding proteins (P value threshold = 5%).

**Network 6** Molecular functions network for significant protein hits (interactors) of G4-1 (both 2.5 and 25 μM concentrations), P value threshold = 5%.

**Network 7** Molecular functions network for G4-1, G4-2 and G4-3 (25 μM concentration) binding proteins (P value threshold = 21%).
