# Supplementary figures and images for: Data set on G4 DNA interactions with human proteins
Source: Data Brief. 2018 Mar 9;18:348–59. doi: 10.1016/j.dib.2018.02.081 (PMC5996148; doi:10.1016/j.dib.2018.02.081)

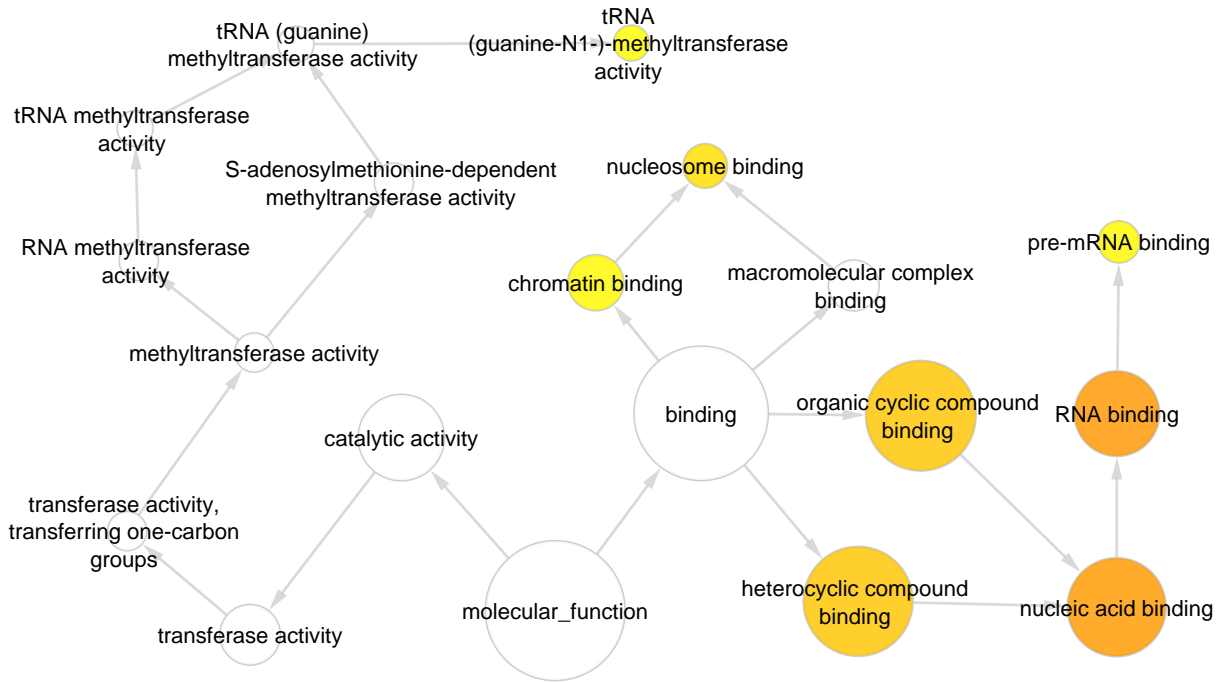

Supplement: Supplementary file 4 — Network 1 Molecular functions network for significant protein hits (interactors) of G4-2 and G4-3 (25 μM concentration), P value threshold = 5%. [file mmc12.pdf]

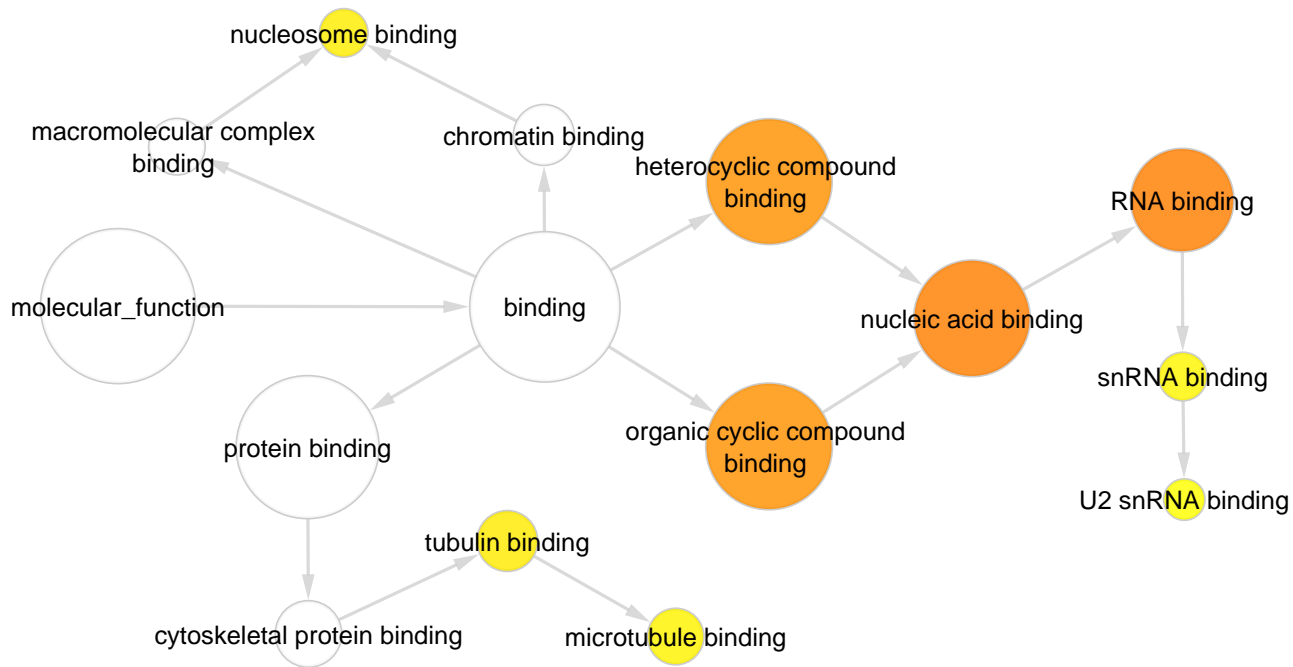

Supplement: Supplementary file 5 — Network 2 Molecular functions network for significant protein hits (interactors) of G4-2 (both 2.5 and 25 μM concentrations), P value threshold = 5%. [file mmc13.pdf]

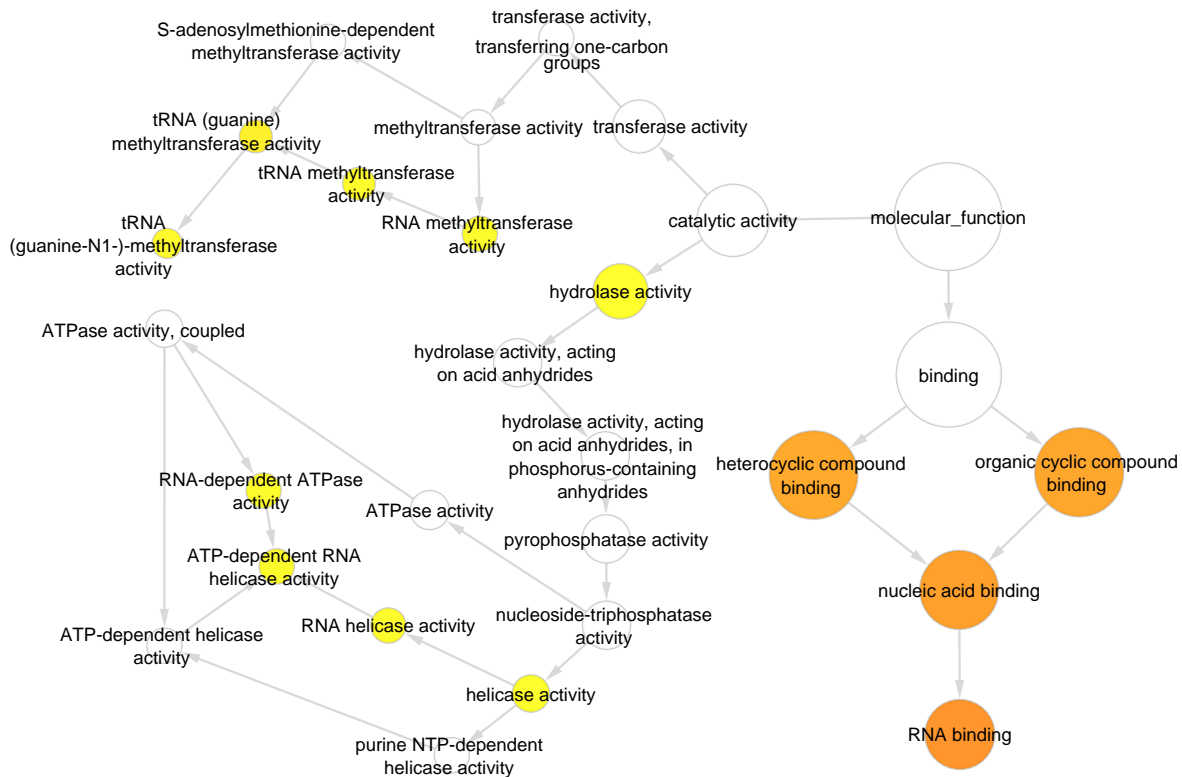

Supplement: Supplementary file 6 — Network 3 Molecular functions network for significant protein hits (interactors) of G4-3 (both 2.5 and 25 μM concentrations), P value threshold = 5%. [file mmc14.pdf]

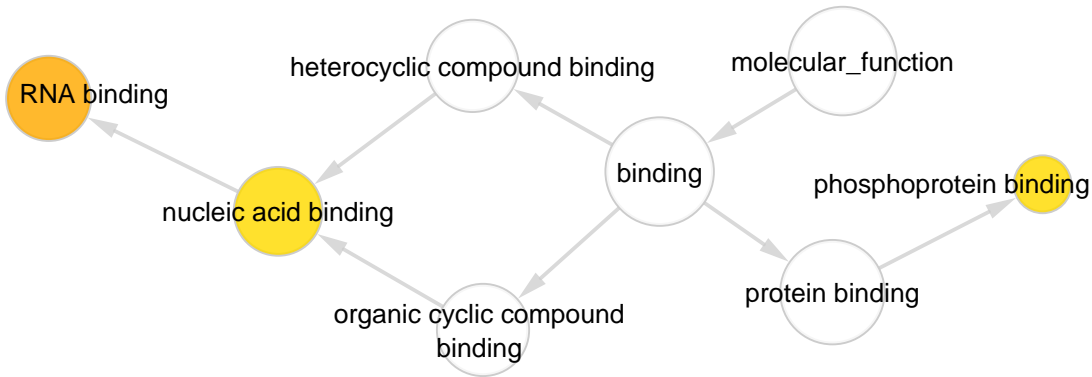

Supplement: Supplementary file 7 — Network 4 Molecular functions network for significant protein hits (interactors) of G4-1 (2.5 μM concentration) binding proteins (P value threshold = 5%). [file mmc15.pdf]

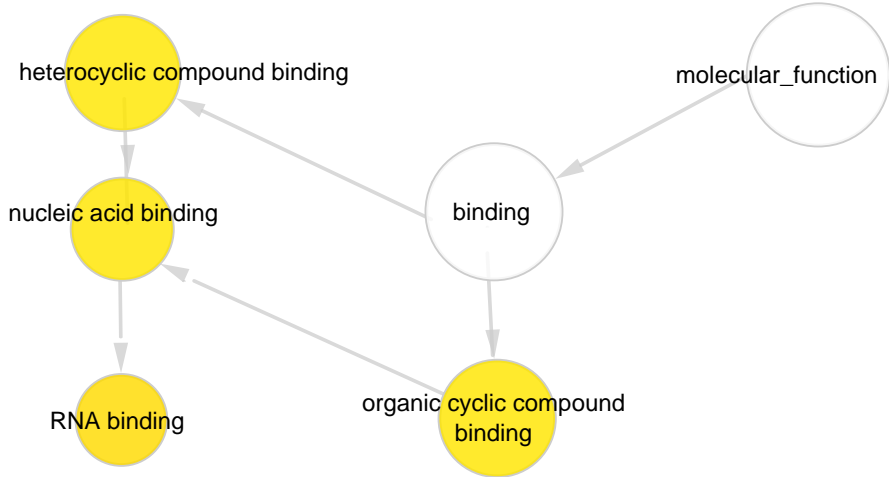

Supplement: Supplementary file 8 — Network 5 Molecular functions network for significant protein hits (interactors) of G4-1 (25 μM concentration) binding proteins (P value threshold = 5%). [file mmc16.pdf]

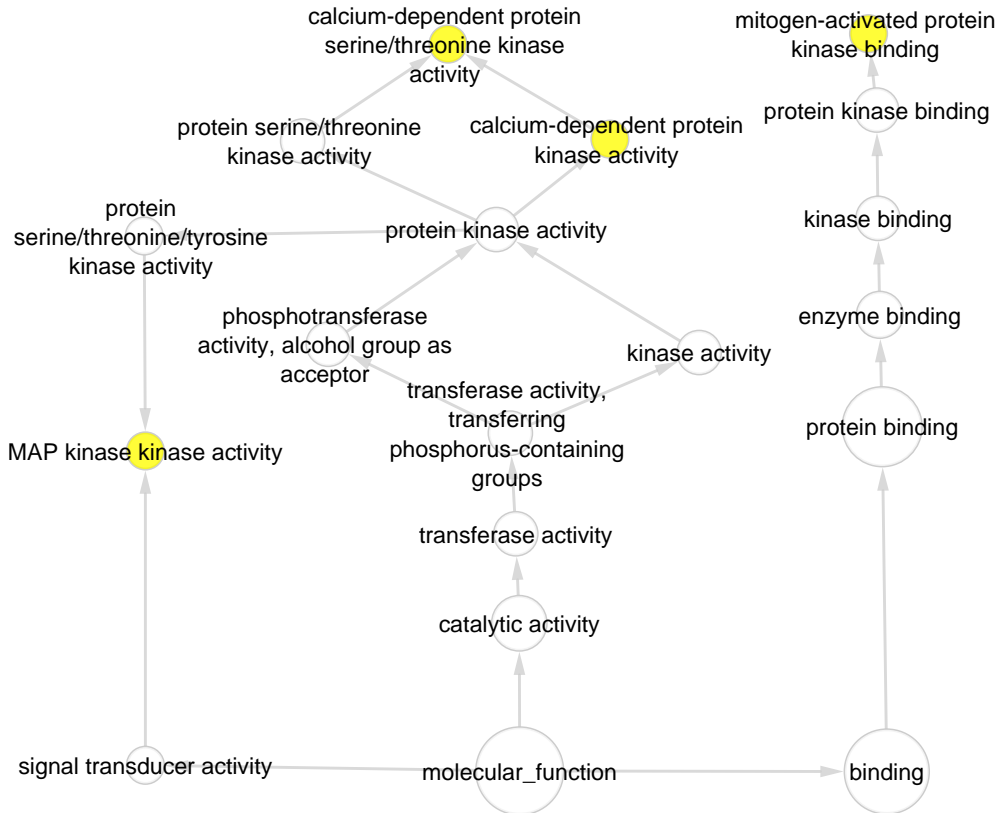

Supplement: Supplementary file 9 — Network 6 Molecular functions network for significant protein hits (interactors) of G4-1 (both 2.5 and 25 μM concentrations), P value threshold = 5%. [file mmc17.pdf]

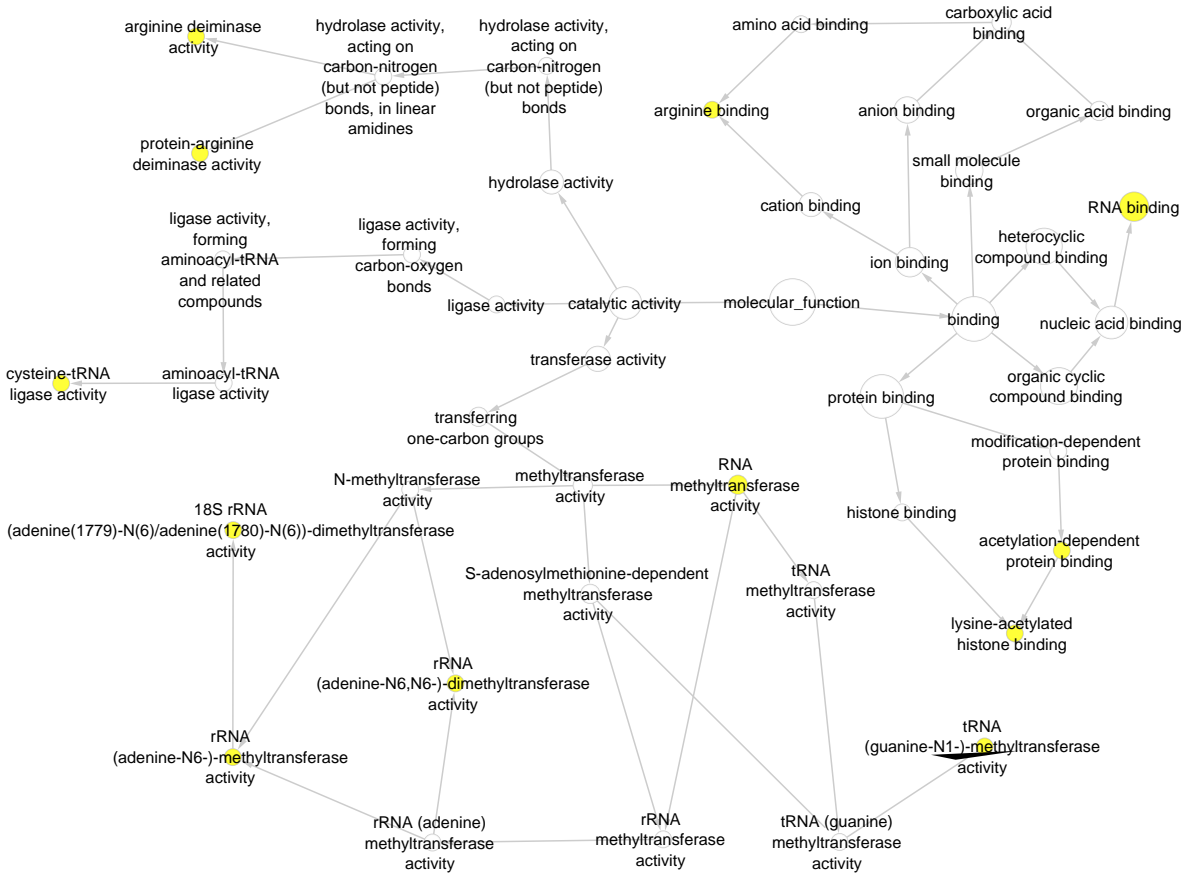

Supplement: Supplementary file 10 — Network 7 Molecular functions network for G4-1, G4-2 and G4-3 (25 μM concentration) binding proteins (P value threshold = 21%). [file mmc18.pdf]
